# Individuals with nonsyndromic orofacial clefts have increased asymmetry of fingerprint patterns

**DOI:** 10.1371/journal.pone.0230534

**Published:** 2020-03-20

**Authors:** Katherine Neiswanger, Nandita Mukhopadhyay, Shwetha Rajagopalan, Elizabeth J. Leslie, Carla A. Sanchez, Jacqueline T. Hecht, Iêda M. Orioli, Fernando A. Poletta, Javier Enríquez de Salamanca, Seth M. Weinberg, Mary L. Marazita

**Affiliations:** 1 Center for Craniofacial and Dental Genetics, Department of Oral Biology, School of Dental Medicine, University of Pittsburgh, Pittsburgh, Pennsylvania, United States of America; 2 Department of Pediatrics, University of Texas McGovern Medical Center, Houston, Texas, United States of America; 3 Laboratory of Congenital Malformation Epidemiology, Oswaldo Cruz Institute, Rio de Janeiro, Brazil; 4 Center for Medical Education and Clinical Research, Estudio Collaborativo Latino Americano de Malformaciones Congénitas, Buenos Aires, Argentina; 5 Sección de Cirugía Plástica, Hospital Infantil Universitario Niño Jesús, Madrid, Spain; 6 Department of Human Genetics, Graduate School of Public Health, University of Pittsburgh, Pittsburgh, Pennsylvania, United States of America; 7 Department of Anthropology, University of Pittsburgh, Pittsburgh, Pennsylvania, United States of America; 8 Clinical and Translational Science, School of Medicine, University of Pittsburgh, Pittsburgh, Pennsylvania, United States of America; Ohio State University, UNITED STATES

## Abstract

Dermatoglyphic patterns on the fingers often differ in syndromes and other conditions with a developmental component, compared to the general population. Previous literature on the relationship between orofacial clefts–the most common craniofacial birth defect in humans–and dermatoglyphics is inconsistent, with some studies reporting altered pattern frequencies and/or increased asymmetry and others failing to find differences. To investigate dermatoglyphics in orofacial clefting, we obtained dermatoglyphic patterns in a large multiethnic cohort of orofacial cleft cases (N = 367), their unaffected family members (N = 836), and controls (N = 299). We categorized fingerprint pattern types from males and females who participated at five sites of the Pittsburgh Orofacial Cleft study (Hungary, United States of America (Pennsylvania, Texas), Spain, and Argentina). We also calculated a pattern dissimilarity score for each individual as a measure of left-right asymmetry. We tested for group differences in the number of arches, ulnar and radial loops, and whorls on each individual’s hands, and in the pattern dissimilarity scores using ANOVA. After taking sex and site differences into account, we did not find any significant pattern count differences between cleft and non-cleft individuals. Notably, we did observe increased pattern dissimilarity in individuals with clefts, compared to both their unaffected relatives and controls. Increased dermatoglyphic pattern dissimilarity in individuals with nonsyndromic orofacial clefts may reflect a generalized developmental instability.

## Introduction

**Fig 1:**
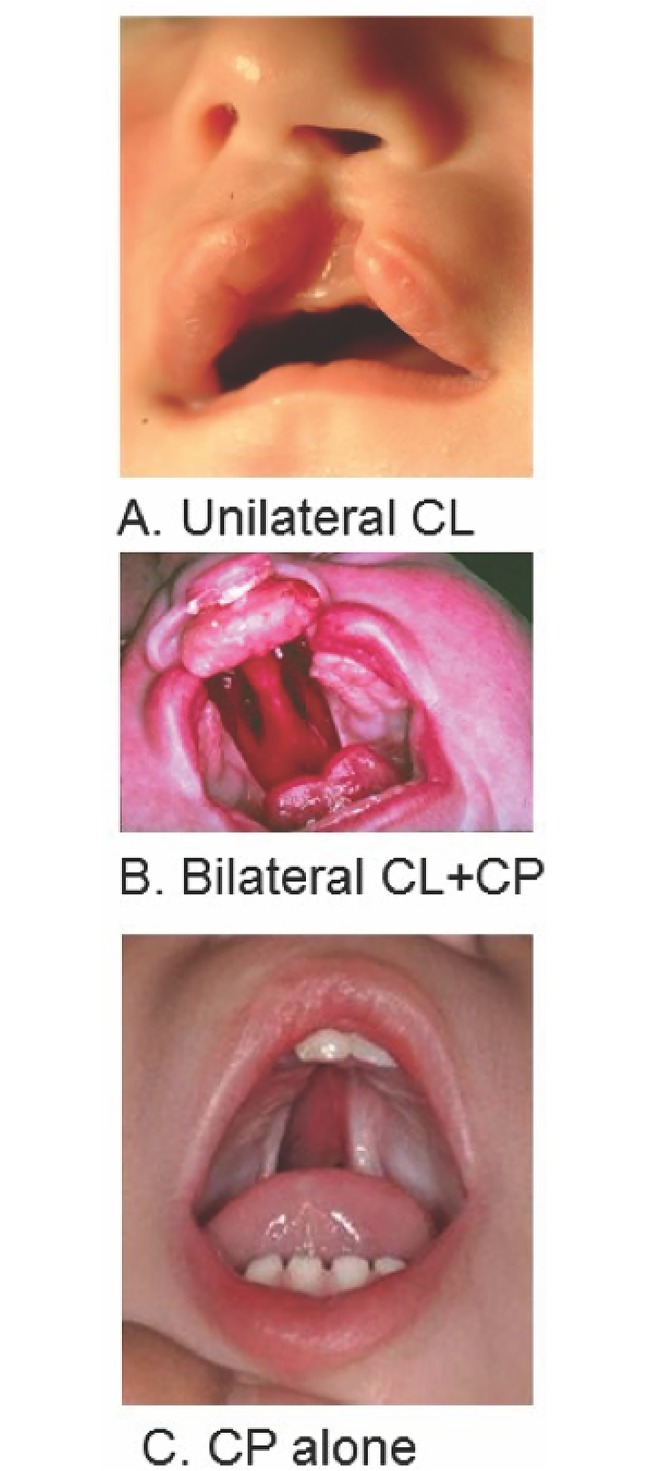
Types of nonsyndromic oral facial clefts. Nonsyndromic clefts can involve the lip only (A), both lip and palate (B), or the palate only (C). They range in severity from small lip notches and submucous cleft palates to the severe case shown in (B). CL = cleft lip; CP = cleft palate.

Orofacial clefts are formed by improper or failed fusion of tissues during early development (Fig 1). They are among the most common birth defects worldwide and can present in isolation or be syndromic [1]. Nonsyndromic cleft lip with or without cleft palate (CL/P) has birth prevalence rates ranging from a high of 1/500 in Asian and Native American populations to lower prevalences of 1/2500 in Africans, while the prevalence of nonsyndromic cleft palate only (CPO) is about half that of CL/P worldwide [1,2]. CL/P and CPO are both complex multifactorial traits, controlled by multiple genes and environmental factors [1–6].

The frequencies of dermatoglyphic patterns on the fingers and palms have been studied for many years as potentially sensitive indicators of generalized prenatal developmental delay or instability [7–10]. Because there exists an embryological overlap in the chronologies of the formation of the lip and palate *in utero* (4th-9th week) [11,12] and dermatoglyphics (6th– 24th week) [13–16], numerous studies have examined dermatoglyphic traits in nonsyndromic clefting in multiple populations. Many have reported altered frequencies of dermatoglyphic patterns [16–30] or increased pattern asymmetry [22,24,27,28,31–36] between cleft cases, unaffected relatives, and/or controls. However, results have been inconsistent, with other studies reporting no dermatoglyphic differences in individuals with orofacial clefts [37–41].

The Pittsburgh Oral Facial Cleft study (POFC) began in 1993 and aims to identify genes for nonsyndromic orofacial clefting through a number of strategies, including deep phenotyping of affected cases, their unaffected relatives, and controls from around the world [42,43]. We hypothesize that, due to the shared embryological chronology between the formation of the lip/palate and fingerprints in the first trimester, individuals with nonsyndromic clefts may show altered dermatoglyphic pattern counts and/or increased pattern asymmetry, when compared to individuals without clefts. Our aim is to test these two hypotheses in the POFC sample by ANOVA analysis of dermatoglyphic pattern counts and asymmetry among individuals with clefts, their unaffected relatives, and controls.

## Materials and methods

The University of Pittsburgh Institutional Review Board approved this study (IRB protocol numbers STUDY19030367 and STUDY19090156). Written consent was obtained from all participants.

### Sample characteristics

The POFC study focuses on identifying genes for nonsyndromic clefting [5,43]. Collaborating clinicians at hospitals, cleft centers, and field sites worldwide refer their nonsyndromic cleft patients and families to the study, or screen potential participants at cleft surgery clinics, to exclude individuals with suspected syndromes, e.g., Van der Woude. Control families and individuals are recruited through advertisements and word of mouth. They are screened for the absence of any cleft, and have no known history of cleft in their first- or second-degree relatives. After obtaining written informed consent from the University of Pittsburgh IRB and other international IRBs or Institutional Ethics Committees, trained research staff collect several additional phenotypes. From 1999–2011, dermatoglyphic prints were collected at five sites internationally (Hungary, Pennsylvania in the U.S.A. (USA-PA), Texas in the U.S.A. (USA-TX), Spain, and Argentina). POFC participants from these five sites with 9 or 10 scoreable fingerprints were included in this study, for a total of 1502 individuals (801 female; 701 male).

All forms of nonsyndromic clefting—cleft lip only, cleft lip and palate, and cleft palate only—were included in this analysis. POFC initially concentrated on nonsyndromic cleft lip.

With or without cleft palate, so there are comparatively few people with cleft palate only. Since alterations in fingerprints are more likely to reflect generalized early developmental instability, as opposed to specific mechanisms of lip or palate formation, we combined individuals with all types of cleft in this analysis. The POFC study is enriched for multiplex cleft families, i.e. those with more than one affected family member, and the sample includes all available family members, with or without a cleft, as well as multiple family members from the control families. [43]. We divided study participants into three groups: 1) Cases– 367 individuals with a nonsyndromic CL/P or CPO; 2) Unaffected Family Members (UFMs)– 836 individuals from case families who did not have a cleft; and 3) Controls– 299 individuals from control families who were recruited at the Hungary and USA-PA sites only.

### Fingerprint collection

**Fig 2:**
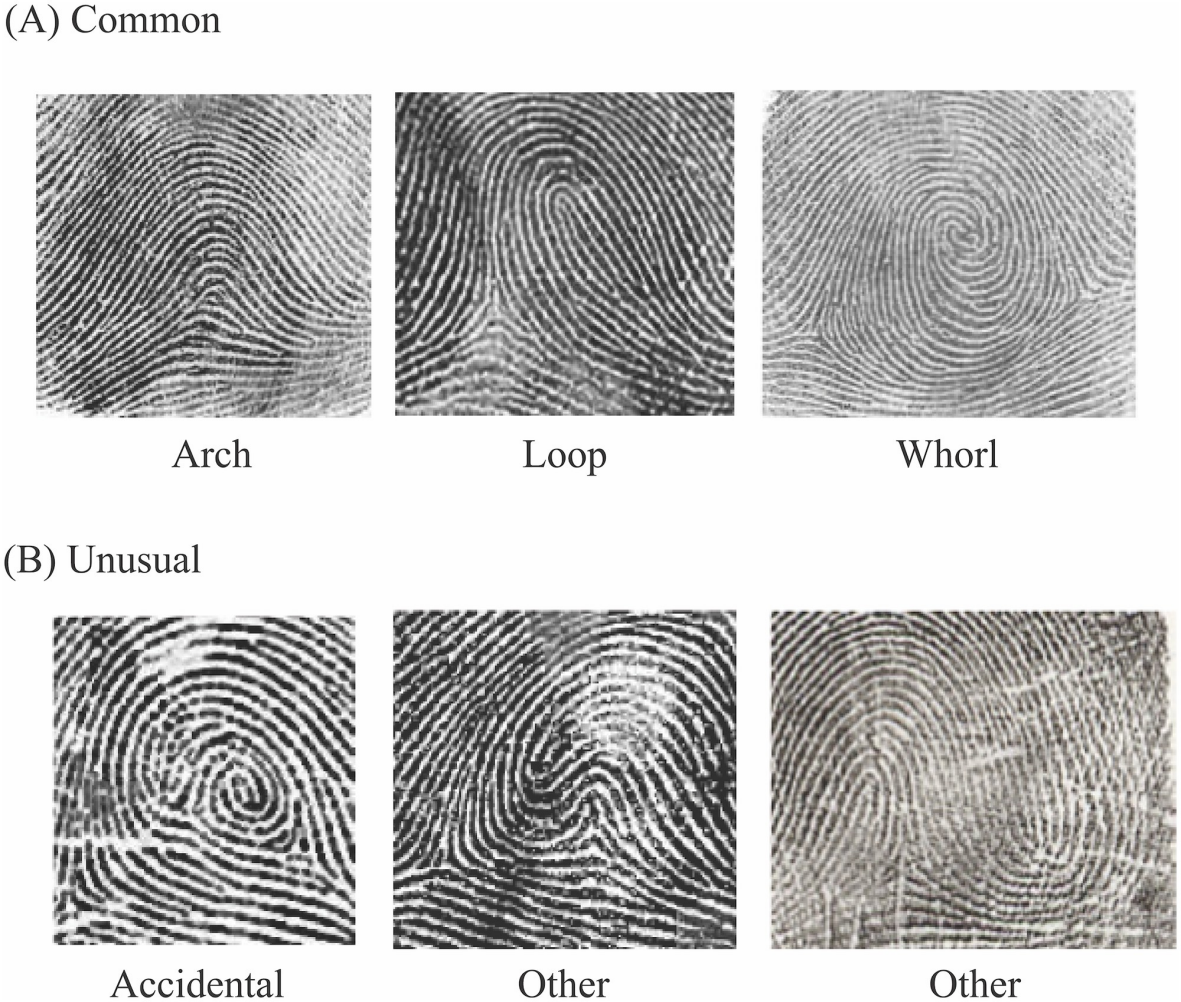
Dermatoglyphic patterns. (A) Arches, loops, and whorls are common fingerprints. Loops can be either ulnar or radial, depending on whether they open to the ulnar or radial side of the finger. (B) Uncommon fingerprints include accidentals and other rare types.

We took fingerprints using the standard ink method [44,45]. Three trained raters each scored all the patterns on every finger as arch (A), ulnar loop (UL), radial loop (RL), whorl (W), or accidental/other uncommon print. All prints that were not A, UL, RL, or W were grouped into a single “Other” (O) category. If the three initial scores did not agree, a fourth, experienced rater re-evaluated the print. Spot-checks were also performed on sub-sets of prints. Only individuals with 9–10 ratable prints were included in this analysis. See Fig 2 for examples of fingerprint patterns from our participants.

### Pattern count ANOVA

We used analysis of variance (ANOVA) to model the joint effects of recruitment site, sex, cleft status, and their interactions on the dermatoglyphic pattern count, defined as the number of common patterns (i.e., A, UL, RL, W) present on the hands of the 1502 individuals in the sample. Each individual was assigned a four-part pattern count score. For example, a score of (5,3,1,1) shows that this person has 5 UL, 3 W, 1 A, and 1 RL on their hands. People can have extreme scores of (10,0,0,0), (0,10,0,0), (0,0,10,0), or different combinations of pattern types. This composite variable totaled 10 for most people, but could total 9 or fewer for those people who were missing a print and/or had rare prints.

ANOVA was run on the full model that included the effects of site, sex, cleft status + interactions on dermatoglyphic pattern count, and compared to all possible nested models, i.e., models that excluded one or more of the independent variables and their interactions. The most parsimonious model, determined by the Akaike Information Criterion (AIC), was selected as the best fitting model. For those variables with more than two categories (e.g., five sites and three cleft statuses), we also used the Tukey’s *post hoc* Honest Significant Differences (HSD) test to determine which sites or cleft statuses were driving the association. The significance threshold for p values was set to 0.05. All statistical analyses were conducted using the R statistical package [46].

Because the cleft status variable did not include controls at three of the sites, we performed two ANOVAs on slightly different samples. First, we used the “All Sites model,” which included all five sites, but only cases and UFMs for the cleft status variable. Second, we ran the “Hungary + USA-PA model,” which included only the Hungary and USA-PA sites, but included all three cleft statuses—cases, UFMs, and controls.

### Pattern dissimilarity ANOVA

Next, we examined whether patterns differed between the left and right hands according to site, sex, and/or cleft status. For this analysis, patterns were assigned to one of four different types—arches, loops (ulnar and radial loops collapsed into one type), whorls, and other patterns (accidentals and other rare patterns collapsed into one type). Only study participants with known patterns on all ten digits were included (N = 1,476). Pattern asymmetry between right and left hands was determined by calculating a dissimilarity score for each individual, following [35]. For each pair of digits, e.g., right and left thumb, we assigned a score of ‘0’ if the pattern type was the same on both digits, and a score of ‘1’ if the pattern types did not match. We then summed the digit scores over all five pairs of digits. An individual’s dissimilarity score could range from 0 (all 5 digit pairs with matching pattern types) to 5 (all 5 pairs of digits with dissimilar pattern types).

As done previously, we conducted ANOVA for the full and nested models including site, sex, cleft status, and their interactions, followed by Tukey’s *post hoc* HSD test for pairwise differences between sites and cleft statuses. The significance threshold for p values was set to 0.05. All statistical analyses were conducted using the R statistical package [46]. ANOVAs were run separately for the “All Sites Model” and the “Hungary + USA-PA Model.”

## Results

### Sample demographics and pattern frequencies

To describe the sample, Tables 1 and 2 provide the number of individuals and the number of common patterns, respectively, by site, sex, and cleft status. Table 1 shows that in the complete sample of 1,502 individuals, the overall male-to-female ratio is close to equal (701 male vs. 801 female (47% vs. 53%)). However, the male-to-female ratios within each cleft status are more skewed. There is a preponderance of males among the cleft cases (210 male vs. 157 female (57% vs 43%)), whereas there are more females than males among the UFMs (377 male vs. 459 female (45% vs. 55%)), and the controls (114 male vs. 185 female (38% vs. 62%)). We observed these sex differences in cleft status for the sites taken separately as well.

**Table 1:** Number of individuals by site, sex, and cleft status.

Cleft Status	Site					Total
	Hungary	USA-PA	USA-TX	Spain	Argentina	
**Cases**	**163**	**81**	**43**	**35**	**45**	**367**
Male	91 (56%)	45 (56%)	29 (67%)	18 (51%)	27 (60%)	210 (57%)
Female	72 (44%)	36 (44%)	14 (33%)	17 (49%)	18 (40%)	157 (43%)
**UFMs**	**392**	**204**	**152**	**82**	**6**	**836**
Male	174 (44%)	91 (45%)	70 (46%)	38 (46%)	4 (67%)	377 (45%)
Female	218 (56%)	113 (55%)	82 (54%)	44 (54%)	2 (33%)	459 (55%)
**Controls**	**124**	**175**	**-**	**-**	**-**	**299**
Male	47 (38%)	67 (38%)	**-**	**-**	**-**	114 (38%)
Female	77 (62%)	108 (62%)	**-**	**-**	**-**	185 (62%)
**Total**	**679**	**460**	**195**	**117**	**51**	**1502**
Male	312 (46%)	203 (44%)	99 (51%)	56 (48%)	31 (61%)	701 (47%)
Female	367 (54%)	257 (56%)	96 (49%)	61 (52%)	20 (39%)	801 (53%)

UFM = Unaffected Family Member

**Table 2:** Number of common patterns by site, sex, and cleft status.

Site	Cleft Status	N	Number (%) of Pattern Types			
			A	UL	RL	W
**Hungary**	**Cases**	**163**	**95 (6%)**	**952 (59%)**	**73 (5%)**	**491 (30%)**
	Male	91	41 (5%)	547 (61%)	37 (4%)	275 (31%)
	Female	72	54 (8%)	405 (57%)	36 (5%)	216 (30%)
	**UFMs**	**392**	**204 (5%)**	**2394 (62%)**	**154 (4%)**	**1130 (29%)**
	Male	174	74 (4%)	1033 (60%)	80 (5%)	534 (32%)
	Female	218	130 (6%)	1361 (63%)	74 (3%)	596 (28%)
	**Controls**	**124**	**60 (5%)**	**770 (63%)**	**49 (4%)**	**348 (28%)**
	Male	47	32 (7%)	280 (60%)	19 (4%)	135 (29%)
	Female	77	28 (4%)	490 (64%)	30 (4%)	213 (28%)
**USA-PA**	**Cases**	**81**	**51 (6%)**	**479 (59%)**	**45 (6%)**	**231 (29%)**
	Male	45	22 (5%)	257 (57%)	25 (6%)	144 (32%)
	Female	36	29 (8%)	222 (62%)	20 (6%)	87 (24%)
	**UFMs**	**204**	**123 (6%)**	**1334 (66%)**	**125 (6%)**	**445 (22%)**
	Male	91	47 (5%)	557 (62%)	73 (8%)	227 (25%)
	Female	113	76 (7%)	777 (69%)	52 (5%)	218 (19%)
	**Controls**	**175**	**101 (6%)**	**1078 (62%)**	**88 (5%)**	**472 (27%)**
	Male	67	37 (6%)	426 (64%)	38 (6%)	166 (25%)
	Female	108	64 (6%)	652 (61%)	50 (5%)	306 (29%)
**USA-TX**	**Cases**	**43**	**30 (7%)**	**287 (67%)**	**17 (4%)**	**92 (22%)**
	Male	29	21 (7%)	197 (69%)	11 (4%)	58 (20%)
	Female	14	9 (6%)	90 (65%)	6 (4%)	34 (24%)
	**UFMs**	**152**	**81 (5%)**	**957 (63%)**	**63 (4%)**	**408 (27%)**
	Male	70	29 (4%)	435 (63%)	34 (5%)	198 (28%)
	Female	82	52 (6%)	522 (64%)	29 (4%)	210 (26%)
**Spain**	**Cases**	**35**	**31 (9%)**	**246 (71%)**	**19 (6%)**	**49 (14%)**
	Male	18	16 (9%)	128 (72%)	11 (6%)	23 (13%)
	Female	17	15 (9%)	118 (71%)	8 (5%)	26 (16%)
	**UFMs**	**82**	**76 (9%)**	**545 (67%)**	**37 (5%)**	**158 (19%)**
	Male	38	39 (10%)	214 (57%)	22 (6%)	103 (27%)
	Female	44	37 (8%)	331 (76%)	15 (3%)	55 (13%)
**Argentina**	**Cases**	**45**	**20 (5%)**	**254 (57%)**	**14 (3%)**	**156 (35%)**
	Male	27	15 (6%)	155 (58%)	7 (3%)	89 (33%)
	Female	18	5 (3%)	99 (56%)	7 (4%)	67 (38%)
	**UFMs**	**6**	**0 (0%)**	**38 (63%)**	**0 (0%)**	**22 (37%)**
	Male	4	0 (0%)	29 (72%)	0 (0%)	11 (28%)
	Female	2	0 (0%)	9 (45%)	0 (0%)	11 (55%)

N = Number of individuals from which pattern frequencies are derived; A = Arch; UL = Ulnar Loop; RL = Radial Loop; W = Whorl; UFM = Unaffected Family Member

Table 2 provides the frequencies of the common dermatoglyphic patterns by site, sex, and cleft status. The 1,502 individuals contributed 14,893 common patterns to Table 2, with 101 rare patterns and 26 missing prints not included. In Table 2, UL is the most frequent pattern, followed by W, A, and RL. Visual inspection of Table 2 suggests that the frequencies of common dermatoglyphic patterns vary by site, sex, and/or cleft status.

### Pattern count ANOVA—Effects of site, sex, and cleft status

The dermatoglyphic pattern counts per individual showed the same general trends as the pattern frequencies in Table 2. UL are present on an average of at least six fingers, with W averaging 2–3.5 fingers, and A and RL averaging less than one finger per individual. Even so, there are a few individuals in the sample with ten arches on their fingers.

Table 3 provides the results of the ANOVA analyses for those nested models which had a p value ≤ 0.05. For each pattern type and model, Table 3 summarizes the most optimal models based on AIC, including overall and pairwise p values, observed group means, and estimated pairwise group differences plus 95% confidence intervals for the pairwise comparisons. In the All Sites Model, for example, the categorical site variable represents five sites; therefore, its overall p value, if significant, is included in addition to the significant pairwise site differences from the Tukey HSD test. Similarly, test outcomes for the cleft status variable, which has three categories in the Hungary + USA-PA Model, include both pairwise and overall test outcomes that are significant.

**Table 3:** Pattern count—ANOVA optimal models with P values ≤ 0.05.

Pattern Type	Optimal Model^1^	P Value	Observed Group Means Gp1, Gp2	Estimated Group Difference and [95% CI]
**UL**	**All Sites Model:** Site + Sex + Sex*Cleft Status			
	Site	0.01		
	Spain–Hungary^2^	0.03	6.76, 6.06	0.73 [0.04–1.42]
	Sex (Female–Male)	0.02	6.34, 6.08	0.32 [0.04–0.60]
	Sex*Cleft Status	0.04		
	Female.UFM–Male.UFM^2^	0.02	6.54, 6.03	0.49 [0.05–0.94]
**RL**	**All Sites Model:** Site + Sex + Sex*Cleft Status			
	Site	0.002		
	USA-PA–Argentina^2^	0.03	0.56, 0.27	0.32 [0.02–0.62]
	USA-PA–Hungary^2^	0.003	0.56, 0.41	0.19 [0.04–0.33]
	Sex (Male–Female)	0.006	0.51, 0.41	0.11 [0.03–0.19]
	Sex*Cleft Status	0.01		
	Male.UFM–Female.UFM^2^	0.001	0.56, 0.37	0.18 [0.05–0.31]
	**Hungary + USA-PA Model:** Site + Sex + Sex*Cleft Status			
	Site (USA-PA–Hungary)	< 0.001	0.56, 0.41	0.15 [0.07–0.24]
	Sex (Male–Female)	0.01	0.53, 0.42	0.11 [0.03–0.19]
	Sex*Cleft Status	0.04		
	Male.UFM–Female.UFM^2^	0.01	0.58, 0.38	0.20 [0.04–0.37]
**W**	**All Sites Model:** Site + Sex			
	Site	<0.001		
	Hungary–Spain^2^	< 0.001	2.90, 1.77	1.15 [0.41–1.89]
	Argentina–Spain^2^	0.001	3.49, 1.77	1.72 [0.50–2.95]
	Hungary–USA-PA^2^	0.04	2.90, 2.50	0.55 [0.02–1.08]
	Argentina–USA-PA^2^	0.05	3.49, 2.50	1.12 [0.01–2.23]
	Sex (Male–Female)	0.03	2.79, 2.54	0.34 [0.04–0.64]
	**Hungary + USA-PA Model:** Site			
	Site (Hungary–USA-PA)	0.01	2.90, 2.50	0.40 [0.09–0.72]

^1^Based on AIC. For each pairwise test, group1 is on the left and group2 is on the right in columns 2 and 4.

^2^Tukey HSD Pairwise Group

GP1 = Group 1; GP2 = Group 2; CI = Confidence Interval; UL = Ulnar loop; RL = Radial loop; W = Whorl; UFM = Unaffected Family Member

Arches did not show any significant pattern count differences by site, sex, or cleft status. For UL, RL and W, we observed significant count differences depending on both recruitment site and sex. Site differences were most significant for W (p < 0.001) using the All Sites Model, but were also significant for UL and RL. The Spanish sample had significantly more UL than Hungary (6.76 vs. 6.06; p = 0.03), and significantly fewer W (1.77 vs. 2.90; p < 0.001). Even though the average overall RL count is < 1 per individual, there were significantly more RL in the USA-PA sample than in Hungary (0.56 vs. 0.41) for both the All Sites Model (p = 0.003) and for the Hungary-USA-PA Model (p < 0.001).

The all-sites ANOVA also found significant sex differences for UL, RL, and W, although they were not as pronounced as the site differences. Females had significantly more UL than males (6.34 vs. 6.08; p = 0.02), while males had significantly more W (2.79 vs. 2.54; p = 0.03) and RL (0.51 vs.0.41; p = 0.006). Thus, individual pattern counts differ by both site and sex in this data set.

After taking site and sex differences into account, the mean number of UL, RL, and W per individual did not differ significantly by cleft status in either the All Sites sample or the Hungary-USA-PA sample. However, for both UL and RL, there appears to a significant interaction between cleft status and sex in the UFMs. Across all five sites, female UFMs have a higher mean number of UL than male UFMs (mean UL females vs. males: 6.54 vs. 6.03; p = 0.02), and a lower frequency of RL (mean RL females vs. males: 0.37 vs. 0.56; p = 0.001). We observed the RL association in the Hungary-USA-PA sample as well (mean RL female UFMs vs. males: 0.38 vs. 0.58; p = 0.01). The addition of controls in the latter analysis did not change the results.

### Pattern dissimilarity ANOVA—Effects of site, sex, and cleft status

Table 4 provides the mean dissimilarity scores by site, sex, and cleft status for the 1476 individuals with complete sets of 10 readable prints, and Table 5 gives the results of the ANOVA. Under the All Sites Model, we observed a significant increase in the mean dissimilarity score of cleft cases compared to UFMs (1.26 vs 1.08; p = 0.01). This observation was consistent within each site and within males and females, although the individual comparisons did not reach significance. For the Hungary-USA-PA Model, individuals with clefts showed significantly more dissimilarity than either the UFMs (mean dissimilarity score cases vs. UFMs: 1.30 vs. 1.08; p = 0.04) or the controls (mean dissimilarity score cases vs. controls: 1.30 vs. 1.08; p = 0.03). For the All Sites Model, males also had significantly higher dissimilarity scores than females (1.20 vs. 1.06; p = 0.02). This difference was observed within all cleft statuses, although it was not significant. No significant sex effects were observed in the Hungary-USA-PA model. In contrast to the pattern count analysis, cleft status was a significant predictor of pattern dissimilarity between left and right hands, along with sex, while there were no observed differences due to recruitment site, nor any interaction effects.

**Table 4:** Mean pattern dissimilarity scores (N) by site, sex, and cleft status.

Site	Cleft Status			Total
	Cases	UFMs	Controls	
**Hungary**	**1.30 (162)**	**1.17 (382)**	**1.04 (120)**	**1.17 (664)**
Male	1.23 (91)	1.21 (169)	1.00 (47)	1.18 (307)
Female	1.39 (71)	1.14 (213)	1.07 (73)	1.17 (357)
**USA-PA**	**1.31 (81)**	**1.02 (202)**	**1.12 (173)**	**1.11 (456)**
Male	1.38 (45)	1.07 (90)	1.27 (66)	1.20 (201)
Female	1.22 (36)	0.98 (112)	1.02 (107)	1.03 (255)
**USA-TX**	**1.26 (43)**	**1.03 (152)**	--	**1.08 (195)**
Male	1.38 (29)	1.10 (70)		1.18 (99)
Female	1.00 (14)	0.96 (82)		0.97 (96)
**Spain**	**1.00 (34)**	**0.90 (80)**	--	**0.93 (114)**
Male	1.22 (18)	1.18 (38)		1.20 (56)
Female	0.75 (16)	0.64 (42)		0.67 (58)
**Argentina**	**1.24 (41)**	**1.00 (6)**	--	**1.21 (47)**
Male	1.48 (25)	0.75 (4)		1.38 (29)
Female	0.88 (16)	1.50 (2)		0.94 (18)
**All sites**	**1.26 (361)**	**1.08 (822)**	**1.09 (293)**	**1.12 (1476)**
Male	1.31 (208)	1.15 (371)	1.16 (113)	1.20 (692)
Female	1.20 (153)	1.02 (451)	1.04 (180)	1.06 (784)

N = Number of individuals in each group that contribute to the mean dissimilarity score; UFM = Unaffected Family Members

**Table 5:** Pattern dissimilarity scores—ANOVA optimal models with P values ≤ 0.05.

Optimal Model^1^	P Value	Observed Group Means Gp1, Gp2	Estimated Group Difference and [95% CI]
**All Sites Model:** Sex + Cleft Status			
Sex (Male–Female)	0.02	1.20, 1.06	0.14 [0.02–0.26]
Cleft Status	0.01		
Case–UFM^2^	0.01	1.26, 1.08	0.16 [0.03–0.28]
**Hungary + USA-PA Model:** Cleft Status			
Cleft Status	0.02		
Case–Control^2^	0.03	1.30, 1.08	0.22 [0.01–0.43]
Case–UFM^2^	0.04	1.30, 1.08	0.19 [0.01–0.37]

^1^Based on AIC. For each pairwise test, group1 is on the left and group2 is on the right in columns 1 and 3

^2^Tukey HSD Pairwise Group

GP1 = Group 1; GP2 = Group 2; CI = Confidence Interval; UFM = Unaffected Family Member

## Discussion

We analyzed fingerprint patterns from 1502 individuals from the POFC Study, to determine if individuals with clefts have altered pattern counts on their hands or increased pattern type asymmetry, compared to their relatives without clefts or to controls. In general, our results revealed little evidence that cleft status was associated with differences in the number of common patterns on an individual’s hands. However, we did find evidence of increased left-right pattern dissimilarity in affected cleft cases, particularly if they were male.

### Differences in pattern frequency by recruitment site and sex

Dermatoglyphic pattern frequencies vary extensively by race and ethnicity [7, 47], and we were not surprised to observe site-specific differences in our sample. Most notably, the overall frequency of whorls in the sample from Spain (18%) was low while the frequencies of ulnar loops (68%) and arches (9%) were high, compared to the other sites, in particular Hungary and Argentina. As demonstrated in Table 6, our Spanish frequencies are relatively extreme, even when compared to other published samples taken from specific regions of Spain [48–50]. Many of the Spanish families in our sample traveled some distance to participate in POFC, and thus they probably do not reflect any single ethnic or regional population. Furthermore, the Spanish sample size of 117 is not exceptionally small. Although fingerprint patterns often vary significantly between relatively close geographical regions and similar ethnicities (see Table 6), the Spanish samples in this study remain unusual, compared to other sites in our study and to other published Spanish samples.

**Table 6:** Frequency of pattern types in spanish samples.

Site	Sex	N	Pattern Type Frequency (%)			
			A	UL	RL	W
**Spain** (current study)						
Male		56	9.89	61.51	5.93	22.66
Female		61	8.60	74.21	3.80	13.39
**Alberche/Tormes Valley** [50]						
Male		187	3.32	58.82	4.12	33.74
Female		219	4.29	61.19	3.06	31.46
**Basque Alava Region, Llanada population** [48]						
Male		99	2.13	62.01	4.04	31.82
Female		82	4.87	61.58	2.68	20.86
**Basque Guipuzcoa Region, Goierri population**						
Male		100	5.40	62.70	4.80	27.10
Female		101	3.37	65.25	4.15	27.23
**Basque Navarra Region, Baztan population**						
Male		92	6.52	61.96	5.87	25.65
Female		66	5.15	71.06	4.09	19.69
**Basque Vizcaya Region, Markina population**						
Male		93	7.31	59.78	4.52	28.39
Female		198	7.58	61.41	3.84	27.17
**Tierra de Campos** [49]						
Male		417	3.95	60.48	4.17	31.03
Female		416	6.06	61.70	3.73	28.47
**La Alcarria** [49]						
Male		339	5.13	60.56	4.84	29.47
Female		314	8.28	64.08	3.47	24.17
**Murcia** [49]						
Male		163	5.23	60.47	4.60	28.11	
Female		184	8.75	65.90	4.28	21.06	

N = Number of males and females at each site; A = Arch; UL = Ulnar Loop; RL = Radial Loop; W = Whorl

The sample had different proportions of males and females in the cleft cases, UFMs, and controls, with 57% males in the cleft cases, 45% males in the UFMs, and 38% males in the controls. This is not unexpected, since nonsyndromic cleft lip with or without cleft palate is more common in males [11,51], while controls, both family derived and unrelated, often include more females. Dermatoglyphic patterns vary between males and females in general [44,52]. We observed an increase in ulnar loops in females, and an increase in radial loops in males, a trend seen in other studies of Spain (Table 6 and [53]). Thus, we included both site and sex as covariates in the analysis.

### Little evidence of association between clefting and specific fingerprint patterns

Fingerprint patterns are formed in early development, overlapping the period of lip and palate formation, and different pattern frequencies may reflect changes in developmental timing [13]. Arches, for example, are considered to be slower forming patterns, and may be seen more frequently in conditions involving developmental delay [54]. Frequencies of dermatoglyphic patterns have been studied in individuals with clefts and their family members or controls for decades. In the majority of reports, the frequency of whorls is decreased in individuals with nonsyndromic clefts and/or their relatives, compared to controls, while the corresponding frequencies of ulnar/radial loops and/or arches is increased, although not all differences are significant or occur in both sexes. This is to be expected, since by definition, a decreased count for one pattern will lead to increased counts in some of the other patterns. These trends have been observed in studies of Japanese [17,23,30], Austrians [29], Indians [16, 18–20,25,26], Israelis [24], Filipinos [28], and Iranians [21,22]. Other studies, however, have failed to replicate these findings, including studies of U.S. Caucasians [41], Belgians [37], Chinese [39], and Indians [38,40].

This study, one of the largest to date, failed to confirm the observation seen in the majority of studies—that whorls are less frequent in individuals with clefts, while loops and/or arches are more frequent. Because the frequencies of both clefting and dermatoglyphic patterns are known to vary significantly in different ethnic groups and by sex, we performed an ANOVA that took all of these factors into account jointly. The analysis compared a full model (site, sex, and cleft status variables) to nested models, and determined that site and sex accounted for most of our observed differences in pattern counts, while cleft status did not. We did observe an interaction between cleft status and sex in the case of radial and ulnar loops. Unaffected male relatives have more radial loops, while unaffected female relatives have more ulnar loops than expected. The biological significance of this observation is not clear, but the implication is that in families with nonsyndromic clefting, the process by which a loop becomes lateralized differs between males and females, but only in individuals who do not have a cleft.

Earlier studies have not taken our analytical approach, so it is possible that not all studies have adequately controlled for the effects of ethnic variation and/or sex. The consistent nature and direction of the majority of findings in the literature (increased loops/arches and decreased whorls in clefting) lends strength to the hypothesis that dermatoglyphic pattern frequencies reflect a developmental delay or perturbation during the formation of the lip and palate that increases the risk of a nonsyndromic cleft. Since our sample sizes are relatively large, this would imply either that these prior studies were flawed, perhaps by low power due to small sample sizes, or that our analysis was overly conservative.

### Fingerprint pattern asymmetry increased in cleft cases

The embryologic development of many bilaterally symmetric anatomic features is presumed to be identical on the left and right sides, whether such features are influenced by genes, the prenatal environment, or some combination of both. This study observed a significant increase in dermatoglyphic asymmetry—measured by a pattern-type dissimilarity score—in individuals with clefts compared to UFMs and controls. We observed an independent increase in pattern dissimilarity in males compared to females. These results were consistent across most sites, indicating that both cleft status and sex are associated with greater dermatoglyphic asymmetry in our sample. However, in Hungary the female cases have more dissimilarity than the male cases, which may explain why the sex effect was not significant in the Hungary-USA-PA model.

Increased asymmetry in multiple dermatoglyphic traits taken from either fingers or palms has been widely reported in different groups of individuals, with or without clefts. Some studies find increased dermatoglyphic asymmetry in individuals with clefts, compared to UFMs and/or controls [24,33]. Others report increased dermatoglyphic asymmetry in cleft individuals and/or their UFMs from multiplex cleft families, compared to either simplex families or controls [31,35,36,39]. There are reports of increased dermatoglyphic asymmetry in parents of individuals with clefts, compared to controls [22 (mothers only), 32 (mothers only), 33,34]. In contrast, [28] reported increased dermatoglyphic asymmetry in female unaffected relatives, compared to cases. Taken together, these studies suggest that dermatoglyphic asymmetry reflects a generalized developmental disturbance that also impacts cleft formation. More specifically, there is evidence of shared genetic mechanisms between limb or appendage developmental processes and cleft formation. For example, a number of cleft-related syndromes include limb defects as well (OMIM search term = “cleft lip/palate and limb”, and a recent GWAS study of human facial shape reported that associated loci were enriched for genes involved in limb development [55]. Whether both phenotypes are the result of mutations in genes for the robustness of early embryonic development or reflections of prenatal environmental insults or an interaction between the two remains to be determined.

### Limitations

Even though our sample sizes are large, recruiting from multiple sites with different racial backgrounds introduced ethnicity as a potential confounder, which could have reduced the power of the sample. Likewise, the decision to define cleft status broadly as CL and/or CP might have introduced additional heterogeneity into the sample, which could also reduce power. Another consideration is that our study sample contains related individuals. Cleft statuses of related individuals may be correlated due to shared genetic as well as environmental factors, the degree of correlation being highest for first-degree relatives (parents, siblings and children). We verified, prior to analysis, that the number of first-degree relatives included from each family is small (2 or 3 for nearly all pedigrees), and that there are no large clusters of correlated subjects in any sub-group. We therefore concluded that the likelihood of observing spurious significant associations due to this correlation is small. The analysis was designed to take these factors into account, but it may have missed small difference in pattern counts, which have been reported in other studies of dermatoglyphics and nonsyndromic clefting. This is countered by the significant laterality differences, in which individuals with clefts showed increased pattern dissimilarity, regardless of recruitment site or sex, showing that there is ample power in this sample for that phenotype. In any event, additional studies in large, well-characterized samples are warranted.

## Conclusions

In this study of nonsyndromic clefting and dermatoglyphic patterns, there were no significant differences in pattern counts for individuals with clefts, their unaffected relatives, or controls. However, individuals with clefts had significantly more dermatoglyphic asymmetry in their pattern types, as measured by a dissimilarity score, than unaffected relatives or controls. These results lend support to the idea that generalized developmental disturbances acting early in pregnancy may increase the risk of orofacial clefting.

## Supporting information

S1 Data (XLSX)
